# Implementing verifiable oncological imaging by quality assurance and optimization (i‑Violin): Protocol for a European multicenter study

**DOI:** 10.1007/s00117-024-01389-8

**Published:** 2024-10-30

**Authors:** Tobias Jorg, Moritz C. Halfmann, Lukas Müller, Fabian Stoehr, Peter Mildenberger, Monika Hierath, Graciano Paulo, Joana Santos, John Damilakis, Ivana Kralik, Boris Brkljacic, Danijel Cvetko, Dimitrij Kuhleji, Hilde Bosmans, Dimitar Petrov, Shane Foley, Paula Toroi, Jonathan P. McNulty, Christoph Hoeschen

**Affiliations:** 1 Department of Diagnostic and Interventional Radiology, University Medical Center of the Johannes Gutenberg-University Mainz, Langenbeckst. 1, 55131 Mainz, Germany; 2 European Institute for Biomedical Imaging Research (EIBIR), Am Gestade 1, 1010 Vienna, Austria; 3 Coimbra Health School, Polytechnic Institute of Coimbra, Rua 5 de Outubro—S. Martinho do Bispo, Apartado 7006, 3046-854 Coimbra, Portugal; 4 School of Medicine, University of Crete, 2208, 71003 Iraklion, Crete Greece; 5 Dubrava University Hospital, Avenija Gojka Suska 6, 10000 Zagreb, Croatia; 6 University Medical Centre Ljubljana, Ljubljana, Slovenia; 7 Medical Imaging Research Center, Department of Radiology, University Hospitals Leuven, Herestraat 49, 3000 Leuven, Belgium; 8 Radiography & Diagnostic Imaging, School of Medicine University College Dublin, Dublin, Ireland; 9 STUK—Radiation and Nuclear Safety Authority, Jokiniemenkuja 1, 01370 Vantaa, Finland; 10 Institute of Medical Technology, Faculty for electrical engineering and Information Technology, Otto-von-Guericke-University Magdeburg, Otto-Hahn-Str. 2, 39106 Magdeburg, Germany

**Keywords:** Imaging standardization, Oncology, Radiation protection, Dosimetry, Education, Training

## Abstract

**Background:**

Advanced imaging techniques play a pivotal role in oncology. A large variety of computed tomography (CT) scanners, scan protocols, and acquisition techniques have led to a wide range in image quality and radiation exposure. This study aims at *i*mplementing *v*er*i*fiable *o*ncological imaging by qua*li*ty assurance and optimizatio*n* (i-Violin) through harmonizing image quality and radiation dose across Europe.

**Methods:**

The 2‑year multicenter implementation study outlined here will focus on CT imaging of lung, stomach, and colorectal cancer and include imaging for four radiological indications: diagnosis, radiation therapy planning, staging, and follow-up. Therefore, 480 anonymized CT data sets of patients will be collected by the associated university hospitals and uploaded to a repository. Radiologists will determine key abdominopelvic structures for image quality assessment by consensus and subsequently adapt a previously developed lung CT tool for the objective evaluation of image quality. The quality metrics will be evaluated for their correlation with perceived image quality and the standardized optimization strategy will be disseminated across Europe.

**Results:**

The results of the outlined study will be used to obtain European reference data, to build teaching programs for the developed tools, and to create a culture of optimization in oncological CT imaging.

**Conclusion:**

The study protocol and rationale for i‑Violin, a European approach for standardization and harmonization of image quality and optimization of CT procedures in oncological imaging, is presented. Future results will be disseminated across all EU member states, and i‑Violin is thus expected to have a sustained impact on CT imaging for cancer patients across Europe.

## Background

Advanced image techniques play a pivotal role in oncology and are fundamental for the diagnosis of tumor diseases but also for treatment planning, staging, and follow-up. Different imaging modalities might be needed, of which computed tomography (CT) scanning is very common [1–4]. Following this, cancer patients are likely repeatedly exposed to radiation during the course of their disease. This issue is especially pressing for young oncologic patients requiring extended follow-up periods after successful treatment. A widely practiced principle to minimize exposure in these patients is to acquire images at a radiation dose that is as low as reasonably achievable (ALARA principle; [5]). However, prior research has shown that the subjective, unstandardized image quality assessment (“reasonable”) has led to substantial differences across Europe in terms of the acquisition parameters influencing radiation dose during imaging studies [6–11].

This can be mostly attributed to the heavy reliance of image quality assessments upon broad subjective metrics such as “overall image quality” and only few objective measurements such as attenuation and signal-to-noise ratios. Despite the existence of more sophisticated objective image quality parameters such as modulated transfer functions and noise power spectra, these are mostly limited to in vitro phantom experiments due to the technical intricacies of the placement of respective regions of interest and complex interactions with iterative reconstruction algorithms [12].^1^

Additionally, most available tools either asses image quality alone or evaluate radiation dose alone, thereby not offering a meaningful approach for optimization of imaging using ionizing radiation. The few currently available software solutions, which allow for an integrated approach (e.g., Qaelum DOSE, SECTRA DoseTrack), document radiation dose on a per-patient and per-scan level and enable simultaneous subjective image quality assessment using Likert scales. However, subjective image quality criteria have only limited reproducibility, and most software solutions have not been cross-validated yet. Thus, the absence of validated tools for (in vivo) image quality assessment and dose evaluation remains a major hurdle on the path to optimization of oncological imaging approaches in clinical practice.

The MEDIRAD (Recommendations on Implications of Medical Low Dose Radiation Exposure) project developed a tool for radiation dose tracking and simultaneous automated objective in vivo image quality assessment for chest CT [13]. The tool is capable of automatically computing modulation transfer functions and noise power spectra of pre-determined clinically relevant regions of interest from in vivo images [14]. Thus, it represents a first step toward bridging the gap between reproducible objective image quality assessment and routine clinical images. However, despite successful development of this tool, it is still in need of a platform for broad dissemination and has to be adapted for determining meaningful image quality parameters directly from the body regions of the abdomen and pelvis.

Therefore, the outlined study will aim to adapt the previously developed tools for quantifiable image quality and dose determination in oncological imaging procedures, to implement and validate them in the hospitals involved in the project, and finally to disseminate the tools and resulting optimized procedures to interested hospitals and healthcare providers in Europe. Thus, the overall aim can be summarized as the *i*mplementation of *v*er*i*fiable *o*ncological imaging by qua*li*ty assurance and optimizatio*n* (i-Violin).

## Materials and methods

### Study design

The 2‑year multicenter implementation study throughout Europe is planned to focus on adult CT imaging of lung cancer, stomach cancer, and colorectal cancer and to include imaging for four indications, i.e., diagnosis, radiation therapy planning, staging, and follow-up. The selection of the three cancer types was based on both the frequency of CT examinations and the frequency of cancers in Europe [15, 16]. i‑Violin will use the tools, reports, and recommendations from the European Commission Horizon 2020 research projects MEDIRAD and SINFONIA (Radiation Risk Appraisal for Detrimental Effects from Medical Exposure During Management of Patients with Lymphoma or Brain Tumour), the Horizon 2020 Coordination and Support Action EURAMED rocc-n-roll (European Medical Application and Radiation Protection Concept), as well as the EUCLID (European Study on Clinical Diagnostic Reference Levels for X‑ray Medical Imaging) project. Unlike the MEDIRAD project, image quality assessment and corresponding dose evaluation will primarily be done for the body regions of the abdomen and pelvis instead of the chest.

In addition, the national competent authority in our consortium will facilitate outreach to experts and authorities for feedback and foster the further implementation beyond the i‑Violin partners and into national legislation, recommendations, or guidelines. There is a dedicated link to the steering group of the SAMIRA (Strategic Agenda for Medical Ionizing Radiation Application) action plan of the European commission, which will regularly evaluate the project progress.

### Study population

The study consortium consists of a multidisciplinary team within ten sites and complementary expertise coming from nine EU member states.

It includes the following five university hospitals: Otto von Guericke-Universität Magdeburg (OvGU), Germany; Universitätsmedizin Mainz (UMC Mainz), Germany; Klinicka Bolnica Dubrava Zagreb (KBDZ), Croatia; Clinical Radiology Institute Ljubljana (UMCL), Slovenica; and the University Hospital of the Katholieke Universiteit Leuven (KUL), Belgium. In addition, the project is further supported by the universities Panepistimio Kritis (UoC), Greece, University College Dublin (UCD), Ireland, and the Coimbra Health School, Instituto Politecnico de Coimbra (IPC), Portugal, as well as the national regulator Säteilyturvakeskus (STUK), Finland, and the coordinator EIBIR, a European research management organization focused on the biomedical imaging field.

### Project milestones

**Fig. 1:**
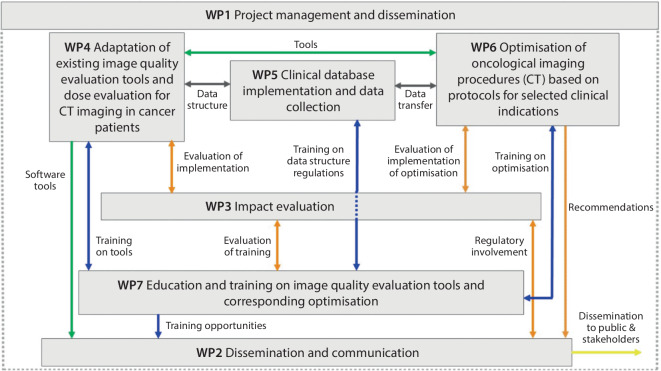
Program evaluation and review technique (PERT) chart of the different work packages of the i‑Violin project including links between them

To achieve the goals of the project, a total of seven work packages with specific objectives and respective leading partner institutions were defined (Table 1). These work packages are strongly interlinked and build upon each other, as displayed in Fig. 1.

**Table 1:** Work packages for i‑Violin

Work package	Title	Lead	Objective
WP 1	Project management and dissemination	EIBIR	Coordination, management and administration, liaison to the SAMIRA action plan and SGQS
WP 2	Dissemination and communication	OvGU	Dissemination, communication of i‑Violin tools as well as education and training programs across EU member states
WP 3	Impact evaluation	UCD	Project evaluation in terms of implementations works
WP 4	Adaption of existing image quality tools and dose evaluation for CT imaging in cancer patients	OvGU	Adjusts the existing image quality and dose evaluation tools for the selected regions of the abdomen and pelvis and validates the tools
WP 5	Clinical database evaluation and implementation	UMC Mainz	Provides the required database infrastructure and collects the required images from the participating clinical sites
WP 6	Optimization of oncological CT protocols for selected clinical indications	KUL	Analysis of the images collected in WP 5 for optimization processes and development of optimized CT protocols
WP 7	Education and training on image quality evaluation tools and corresponding optimization	IPC	Development of education and training programs for radiologists, radiographers and medical physicists

For abbreviations, see text

### Sample size calculation

A total of 480 de-identified and anonymized CT scans of patients with lung, stomach, and colorectal cancer will be retrospectively identified by the university hospitals in the consortium and uploaded to the repository. Inclusion criteria are the confirmed presence of lung, colon, or stomach cancer. For lung cancer, all CT scans for diagnosis, radiation therapy planning, staging, and follow-up are included, while for stomach and colorectal cancer, only examinations for staging and follow-up will be included.

i‑Violin will only deal with fully anonym-ized patient data, to avoid any ethical issues. However, each participating clinical site will inform their ethical review board. Ethics approvals will be obtained from the appropriate bodies prior to starting i‑Violin image collection activities according to the requirements of the respective institutions.

### Image analysis

During the conduction of the study, radiologists at each center will be asked to select one or two structures in the abdominal and pelvic region to characterize the overall quality of the CT scan based on clinical needs and taking into account lessons learned from the MEDIRAD project. Consensus between sites will be reached on the basis of the DELPHI method [17]. These agreed-upon structures will then be used to adapt the MEDIRAD algorithm for abdominal and pelvic imaging.

Subsequently, 30 image studies with varying acquisition parameters and therefore varying image quality levels will be selected from the original dataset of 480 studies and will be evaluated by the same radiologists to judge whether the chosen structures indeed represent different image quality in the images. This subjective rating will be accompanied by applying the adapted MEDIRAD tool for image quality. Ratings of both the subjective rating and the results of the software tool will then be correlated to evaluate their performance and agreement on images that are consensually perceived to be of good and bad image quality.

After internal validation, the tool will then be trained to retrieve the body region as well as the indication for imaging (diagnostic, therapy planning, staging, follow-up) from the CT study protocol and will be applied for the evaluation of image quality of all 480 image studies in the repository.

For optimization of the oncological CT procedures, it is required to determine not only the necessary and achieved image quality but also the corresponding dose distributions. i‑Violin will use commercially available patient dose calculation tools such as DOSE (Qaelum), Radimetrics, or Sectra and then compare the output for chest imaging with the CTRAD software developed in MEDIRAD to validate the commercially available software tools and to quantify deviations. This will again be done for the different imaging indications. For the body regions of the abdomen and pelvis, we will use the output of the commercial dose evaluation tool, but taking into account the uncertainties as derived from the comparison with CTRAD for chest CT.

### Monitoring

i‑Violin has a specific work package dedicated to impact evaluation under the lead of University College Dublin. It will evaluate the implementation of the optimization tools, the optimization, the harmonization, and the standardization of procedures throughout Europe over the time of the project and estimate the impact of the project from the implementation of the tools in hospitals. In addition, it will include the analysis of the factors influencing implementation and the contribution of the i‑Violin project to EU policy objectives (in particular Europe’s Beating Cancer Plan and SAMIRA; [18, 19]).

The status of their achievement is discussed during the monthly management board meetings and a strategy for remedial action is agreed in the case of delays or deviations.

## Results

As of April 2023 there were no preliminary results for i‑Violin. The expected results include a consensus decision of relevant abdominopelvic structures to be used for applying the quality assessment tool from MEDIRAD and the image quality and dose distributions of the 480 CT scans under investigation. Based on these results, proposals for the optimization and harmonization of CT scan protocols will be developed and implemented. The assessment tools and scan protocols will be disseminated across Europe, and teaching courses to ensure a correct usage will be held. This should lead to a culture of optimization in oncological imaging from which all patients could benefit.

## Discussion

This study protocol outlines the methodology for the i‑Violin study, a European multicenter study with a goal to harmonize and standardize CT imaging practices for cancer patients across Europe in order to ensure that radiation dose and image quality are verifiable and well balanced and there is equal access to high-quality healthcare. It represents a concerted effort to evaluate oncological imaging in Europe taking into account the high degree of multidisciplinarity that is needed to reconcile interests from cancer patients, hospitals/cancer centers and their health professionals, national regulators, professional societies at European and national levels, and industry.

A concrete and recent example of this concerted effort needed to confidently evaluate new technical developments by industry partners in hospitals in order to quickly implement innovations into immediate patient care can be seen with the advent of the photon-counting-detector CT systems (PCD-CT). Over the past decade, the established energy-integrating-detector CT seems to have reached in some way its limit in terms of noise as well as spatial and temporal resolution assuming meaningful dose constraints. However, the novel PCD-CT, which has recently been made clinically available by commercial retailers and is being actively scientifically evaluated by healthcare professionals, might hold the potential to overcome these limitations.

Rather than compiling X‑ray quanta and converting them into flashes of light for registration, individual photons are counted and sorted by their energy, resulting in the unprecedented potential to reconstruct images in multiple spectral energy levels from a single CT scan [20]. This and the increased spatial resolution have sparked a great effort by the scientific community to evaluate image quality and establish optimal imaging protocols for different diseases with the goal of improved radiation protection and better optimized CT imaging procedures for patients [21, 22]. The quality metrics developed in the i‑Violin project should be applicable to both the routine CT scanners as well as any emerging CT technology and can provide guidance about the optimization process, including image quality and dose evaluation for healthcare professionals. Thus, the study has the potential to overcome reliance on subjective measurements for relevant aspects such as lesion conspicuity [23]. This verifiable approach can then allow professionals to be more assured about the quality of their images and correspondingly be more confident in their diagnosis or treatment decisions This in turn will impact policymakers at EU and national levels to utilize the developed guidelines and corresponding recommendations as a basis to regulate the requirements regarding CT procedures and ensure equal access.

### Embedment in prior research

The i‑Violin directly profits from a solid foundation of prior evidence-based research such as theEURAMED rocc-n-roll project [24], which identified the key translational challenges for the implementation of radiation protection research into clinical practice. These included system interoperability differences, resource insufficiency, lack of training and education, and the need for greater public awareness surrounding the benefits, risks, and applications of ionizing radiation [25]. These challenges are therefore directly addressed by WP 2 (dissemination and communication), WP 4 (adaptation of existing tools), and WP 7 (education and training) within the i‑Violin project.

Additionally, the MEDIRAD project [14, 26], which aimed at assessing image quality objectively, implementing personalized dosimetry and effectiveness for three-dimensional imaging, will aid in the documentation of standardized procedures and the linking of patient registries in the project.

The tools for comprehensive risk assessment of detrimental effects of radiation exposure, which were developed for chest CT scans within the SINFONIA project [27–30], will also be adapted and further devolved in WP 6 (optimization of oncological CT protocols for selected clinical indications) and thus support the i‑Violin project. Notably, all three projects also involve intensive education and training in medical radiation applications as well as quality and safety standards.

### Limitations and risks

The study design has limitations and potential risks. The latter are assessed for their degree of severity and discussed with potential risk-mitigation strategies. First, due to the study design, the developed tools for harmonization and optimization of image quality and radiation dose levels will be developed on a retrospectively identified cohort of patients and warrant further, prospective, validation in a clinical setting after the dissemination. Second, despite the involvement of nine European countries, this multicenter study does not involve countries such as France, Italy, and Spain, which might be a limiting factor of the heterogeneity of CT scanning protocols and thus underestimate the overall variation in this regard. The risks for the success of the study include the potential absence of a critical team member during the project due to illness (low risk, which is met by the appointment of a deputy for each work package); a sustained or aggravated pandemic could potentially prevent meetings and project-related activities in hospitals (medium risk, which is met by online meetings and online trainings; however, local fine-tuning will be required at a later stage); difficulties in the dissemination of project achievements in CT protocol standardization (medium risk because of the key role in the project, met by the early involvement of professional societies and national regulators in project activities); and lastly problems in data collection and upload by clinical partners (medium risk, met by a backup plan to collect more studies from a different clinical site).

## Conclusion

The study protocol outlined for the i‑Violin project provides a European approach for standardization and harmonization of image quality and optimization of CT procedures in oncological imaging. Through the development, validation, and dissemination of image quality and radiation dose assessment tools for oncological imaging across Europe, it will allow professionals to be more sure about the quality of their images and correspondingly be more confident in their diagnosis or treatment decisions. The tools will be promoted by liaising with key stakeholders in academia, medical professional societies, and national competent authorities. Thus, it is expected to have a sustained impact on CT imaging for cancer patients and lead to a culture of standardized imaging practices across Europe, ensuring equal access to high-quality healthcare.

## Data Availability

The data acquired in the i‑Violin study will be made available upon reasonable request to the study team.

## Footnotes

1 Abbreviations: ESR, European Society of Radiology; EFRS, European Federation of Radiographer Societies; EFOMP, European Federation of Organisations For Medical Physics; EIBIR, European Institute for Biomedical Imaging Research; EUCLID, European study on clinical diagnostic reference levels for x‑ray medical imaging; EURAMED, rocc-n-roll European Medical Application and Radiation Protection Concept; i‑Violin, Implementing verifiable oncological imaging by quality assurance and optimization; MEDIRAD, Recommendations on Implications of Medical Low Dose Radiation Exposure; PERT, Program evaluation and review technique; SAMIRA, Strategic Agenda for Medical Ionizing Radiation Application; SGQS, Steering Group on Quality and Safety of medical applications of nuclear and radiation technology; SINFONIA, Radiation risk appraisal for detrimental effects from medical exposure during management of patients with lymphoma or brain tumour.

## Declarations

i‑Violin will only deal with fully anonymized patient data, to avoid any ethical issues. However, each participating clinical site will inform their ethical review board. Ethics approvals will be obtained from the appropriate bodies prior to starting i‑Violin image collection activities according to the requirements of the respective institutions. The study will be performed in accordance with the ethical standards laid down in the 1964 Declaration of Helsinki and its later amendments.

The supplement containing this article is not sponsored by industry.
